# An Anatomical Study on the Tibial Nerve Bifurcation Level in Relation to the Malleolar-Calcaneal Axis and Its Clinical Significance

**DOI:** 10.7759/cureus.86511

**Published:** 2025-06-21

**Authors:** Dimple Dev V, Suman U

**Affiliations:** 1 Department of Anatomy, Amrita School of Medicine, Amrita Institute of Medical Sciences, Amrita Vishwa Vidyapeetham, Kochi, IND; 2 Department of Anatomy, Kempegowda Institute of Medical Sciences, Bengaluru, IND

**Keywords:** anatomical variation, cadaveric dissection, malleolar-calcaneal axis, posterior leg anatomy, surgical anatomy, tarsal tunnel, tibial nerve

## Abstract

Objective: The objective of this study was to examine the level at which the tibial nerve (TN) bifurcates about the malleolar-calcaneal axis (MCA), a key anatomical reference approximating the inferior margin of the flexor retinaculum, to document anatomical variations with potential clinical and surgical significance.

Methodology: A descriptive cross-sectional study was conducted on 60 lower limbs obtained from formalin-embalmed adult human cadavers. Following standard anatomical protocols, dissections were performed to trace the TN from its origin to its terminal branches. The medial malleolus and the medial tubercle of the calcaneus were identified as landmarks defining the MCA. Based on the nerve’s relationship to this axis, the bifurcation was categorized into three types: type I (proximal to the MCA), type II (at the MCA), and type III (distal to the MCA). Additional observations included morphological variations and the measured distance of the bifurcation point from the MCA.

Results: The bifurcation of the TN was observed above the MCA (type I) in 49 (81.7%) specimens, at the MCA (type II) in nine (15%) specimens, and below the MCA (type III) in two (3.3%) specimens. Regarding the pattern of termination, classical bifurcation (type A) was found in 54 (90%) specimens, trifurcation or the presence of an accessory branch (type B) in five (8.3%) specimens, and no visible bifurcation (type C) in one (1.7%) specimen. The mean distance of bifurcation from the MCA was 15.2±3.4 mm above the MCA and 6.3±1.9 mm below the MCA.

Conclusion: The TN most commonly bifurcates above the MCA, that is, within the tarsal tunnel. This anatomical consistency is clinically relevant during surgical procedures such as tarsal tunnel release and nerve block administration. However, anatomical variants such as trifurcation or accessory branches are not uncommon and must be anticipated to avoid iatrogenic injury. Accurate knowledge of TN bifurcation patterns and their relationship to the MCA is essential for clinical practice surgeons, anesthesiologists, and radiologists.

## Introduction

The tibial nerve (TN), which emerges from the anterior rami of the L4 to S3 spinal nerves, is one of the two terminal branches of the sciatic nerve. It usually separates from the sciatic nerve at the upper angle of the popliteal fossa and travels through the posterior compartment of the leg. It passes underneath the gastrocnemius and soleus muscles. The TN provides motor innervation to several muscles in the leg’s posterior compartment, including the gastrocnemius, soleus, plantaris, popliteus, tibialis posterior, flexor digitorum longus, and flexor hallucis longus, allowing for plantarflexion, inversion, and toe flexion [1,2].

The TN carries both motor function and sensory innervation. It gives off cutaneous branches that innervate the back of the leg and the plantar surface of the foot. The sural nerve, an important sensory branch, receives fibers from both the tibial and common fibular components of the sciatic nerve and innervates the lateral side of the ankle and foot [3]. As the TN approaches the ankle, it passes beneath the flexor retinaculum and posterior to the medial malleolus. It subsequently goes through the tarsal tunnel, a limited fibro-osseous space formed by the medial malleolus, talus, and calcaneus, with the flexor retinaculum serving as its superior limit. Within this tunnel, the nerve typically divides into two terminal branches: the medial plantar nerve (MPN) and the lateral plantar nerve (LPN), which innervate the intrinsic muscles and the skin of the plantar aspect of the foot [4]. The MPN, often the more important of the two, innervates muscles such as the abductor hallucis, flexor hallucis brevis, flexor digitorum brevis, and first lumbrical. It also provides cutaneous innervation to the medial three-and-a-half toes. The LPN innervates the quadratus plantae, abductor digiti minimi, flexor digiti minimi brevis, adductor hallucis, and interossei muscles [5,6].

Tarsal tunnel syndrome is a well-known entrapment neuropathy that results from TN compression within the tarsal tunnel. Patients typically report discomfort, numbness, or tingling in the foot and ankle region. Efficient localization and therapy of this problem requires a good understanding of the TN’s anatomy, particularly its branching pattern. Furthermore, understanding the nerve’s anatomical route is critical for reducing the risk of iatrogenic injury during ankle-related surgical procedures such as fracture repair, arthroscopy, or tendon transfer [7].

Several anatomical studies have explored the branching pattern and classification of the TN within the tarsal tunnel. Havel et al. [8] and Dellon & Mackinnon [9] described variations in the bifurcation site relative to the flexor retinaculum, classifying them into intratunnel, supratunnel, and infratunnel types. Bilge et al. [10] further proposed a classification based on the distance from the malleolar‑calcaneal axis (MCA), highlighting the variability in the nerve’s course. These classification systems emphasize the importance of anatomical landmarks such as the MCA and flexor retinaculum in predicting the likely location of the bifurcation, which is of practical relevance during surgical exposure and nerve block procedures [8-10].

Numerous anatomical studies have revealed variations in the TN’s path, branching patterns, and the location of its terminal bifurcations [8-10]. Such variability of the nerve may impact clinical diagnosis and surgical planning, particularly when accessory branches are present, complicating nerve entrapment syndromes and increasing the risk of intraoperative nerve injury [8]. The MCA, which runs from the apex of the medial malleolus to the medial tubercle of the calcaneus, is a typical anatomical reference for determining the bifurcation of the TN. Prior research has yielded inconsistent results, with some studies indicating that the nerve bifurcates close to the MCA and others reporting division at or beyond this region [6,8,9]. These disparities highlight the need for additional anatomical study to increase our understanding of variability and its therapeutic repercussions.

This study aimed to determine the anatomical location of the TN bifurcation relative to the MCA using human cadaveric lower limb dissections. The fundamental hypothesis was that the degree of bifurcation would differ between individuals, potentially impacting the region’s diagnosis and surgical treatments.

## Materials and methods

Study design

This descriptive cross-sectional study was conducted on 60 formalin-fixed lower limbs of adult human cadavers. The specimens were obtained from the Department of Anatomy at Kempegowda Institute of Medical Sciences and affiliated medical colleges in Bangalore, Karnataka.

Inclusion and exclusion criteria

The study included formalin-fixed lower limbs of adult cadavers, regardless of sex or laterality. Only limbs free from congenital or acquired deformities and without visible signs of traumatic injury were selected for dissection. Limbs showing evidence of prior surgical intervention, trauma, or deformities involving the posterior compartment of the leg or the tarsal tunnel region were excluded to eliminate potential pathological alterations that could affect the anatomical course and branching of the TN.

Dissection procedure

**Figure 1:**
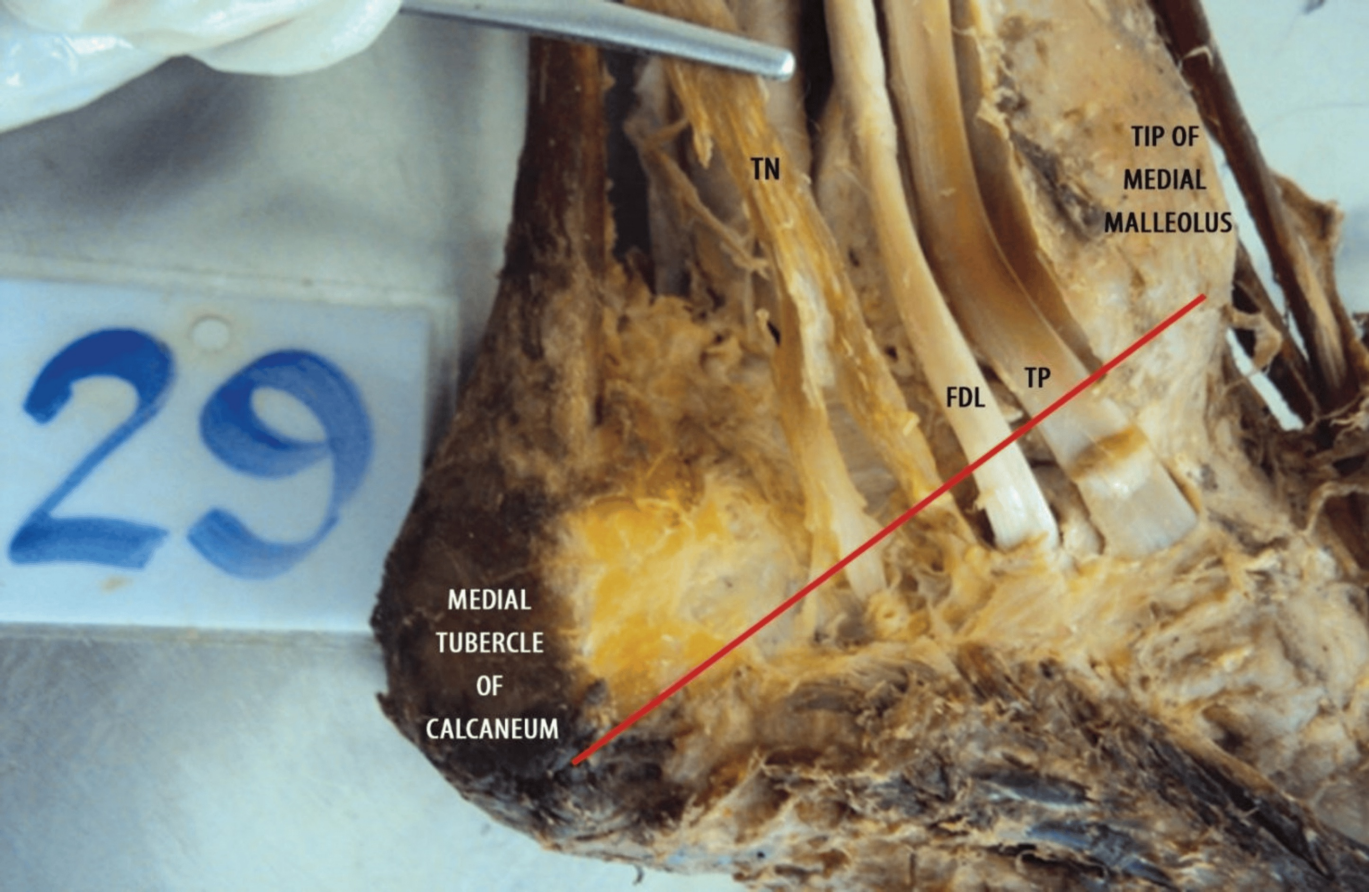
Specimen showing type I pattern of termination of the TN (above the level of MCA) The red line shown in the figure represents the anatomical reference axis extending from the tip of the medial malleolus to the medial tubercle of the calcaneum. TN, tibial nerve; FDL, flexor digitorum longus tendon; TP, tibialis posterior tendon; MCA, malleolar‑calcaneal axis

**Figure 2:**
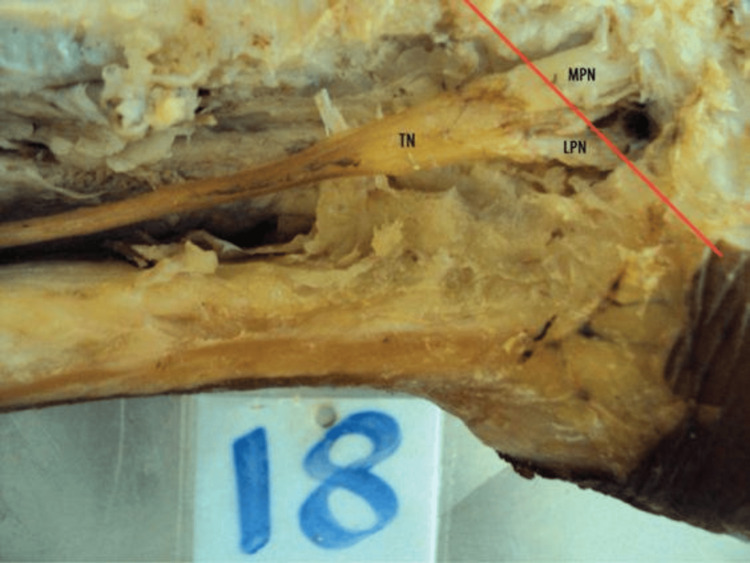
Specimen showing type II pattern of termination of the TN (at the level of MCA) The dissection image shows the origin and branches of the TN. The MPN and LPN are seen branching from the TN in the posterior compartment of the leg at the axis. The red line shown in the figure represents the anatomical reference axis extending from the tip of the medial malleolus to the medial tubercle of the calcaneum. TN, tibial nerve; MPN, medial plantar nerve; LPN, lateral plantar nerve; MCA, malleolar‑calcaneal axis

**Figure 3:**
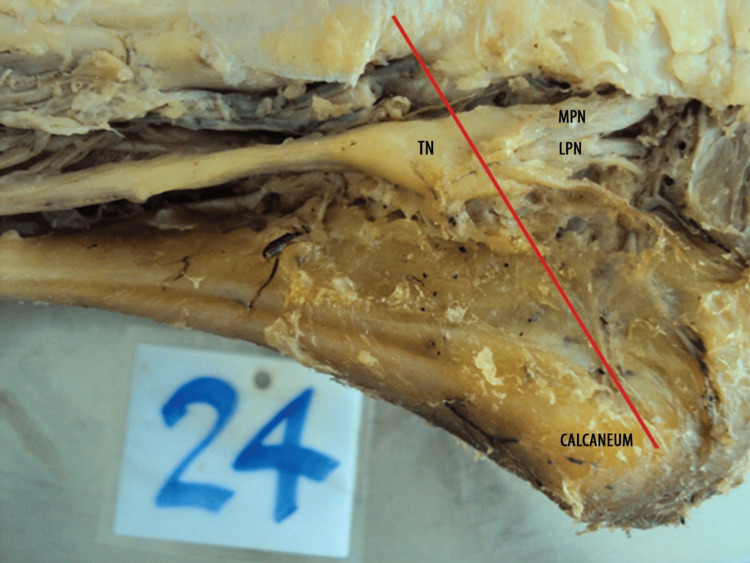
Specimen showing type III pattern of termination of the TN (below the level of MCA) The red line shown in the figure represents the MCA, a clinically significant anatomical reference extending from the tip of the medial malleolus to the medial tubercle of the calcaneus. This axis assists in identifying the branching pattern and orientation of the TN as it divides into the MPN and LPN below it. TN, tibial nerve; MPN, medial plantar nerve; LPN, lateral plantar nerve; MCA, malleolar‑calcaneal axis

Each specimen was dissected according to the standard protocol in Cunningham’s Manual of Practical Anatomy, 15th Edition, Volume 1. The TN was identified and traced from its origin in the popliteal fossa to its terminal bifurcation into the MPN and LPN. Care was taken to preserve the nerve’s integrity and avoid any iatrogenic division, particularly in its distal part. In all specimens presented in Figure 1, Figure 2, and Figure 3, the flexor retinaculum was carefully incised and removed during dissection to allow clear visualization of the tarsal tunnel and the bifurcation of the TN. This approach accurately identified the nerve's branching pattern with respect to the MCA.

Reference axis and anatomical landmarks

The MCA was used as a reference to analyze the bifurcation type of the TN. This axis is a straight line extending from the tip of the medial malleolus to the medial tubercle of the calcaneum. These anatomical landmarks were chosen for their prominence, ease of identification, and proximity to the flexor retinaculum, which forms the roof of the tarsal tunnel. Based on the relationship of the bifurcation point to the MCA, three types of bifurcation were identified: in type, I, the bifurcation occurred above the MCA; in type II, it happened at the level of the MCA; and in type III, it occurred below the MCA.

Documentation and measurement

All specimens were carefully examined, and findings were systematically recorded. Each measurement was taken twice to ensure accuracy, and the average of the two readings was considered the final value to minimize observer and instrument-related errors. High-resolution photographs were taken of each dissected specimen to document the anatomical details of the TN and its branches.

Distribution of bifurcation types

During the dissection and documentation process, the bifurcation type of the TN was recorded for each of the 60 cadaveric lower limbs examined. Based on the position of the bifurcation relative to the MCA, type I bifurcation, where the TN divided above the MCA, was observed in 49 specimens. Type II bifurcation, occurring at the level of the MCA, was noted in nine specimens. Type III bifurcation was found below the MCA and was identified in two specimens. This classification formed an integral part of the methodological assessment aimed at documenting anatomical variations in the branching pattern of the TN (Figures 1-3).

## Results

The present study was conducted on 60 formalin-fixed lower limbs of adult human cadavers to analyze the level of termination of TN in relation to the MCA. In 49 (81.7%) of the specimens, the TN terminated above the MCA, indicating that the most common site of bifurcation lies deep to the flexor retinaculum, i.e., within the tarsal tunnel (Figure 1). This suggests that during surgical interventions involving the tarsal tunnel (e.g., tarsal tunnel release), particular care must be taken to avoid injury to the terminal branches of the TN, which often bifurcate in this region. In nine (15%) cases, bifurcation occurred precisely at the MCA, corresponding to the inferior margin of the flexor retinaculum. This intermediate group represents a transition point where the nerve might still be compressed or involved in tarsal tunnel pathology (Figure 2). Only two (3.3%) specimens demonstrated termination below the MCA, indicating a more distal branching pattern (Figure 3). This rare variant is important to note during plantar approaches or deeper dissections into the sole, where nerve branches may appear lower than anticipated (Table 1). 

**Table 1:** Level of termination of TN in relation to MCA Data are presented as numbers (N) and percentages (%). MCA: malleolar-calcaneal axis; TN, tibial nerve

Level of termination	Description	Number of specimens (N=60)
Level I (above MCA)	Proximal to MCA	49 (81.7%)
Level II (at MCA)	On the MCA	9 (15.0%)
Level III (below MCA)	Distal to MCA	2 (3.3%)

Although the study did not discriminate based on side, the laterality of the limbs dissected was noted for internal comparison. Of the 60 specimens examined, 30 (50%) were from the right side and 30 (50%) were from the left.

During the dissection, care was taken to preserve anatomical integrity. These findings suggest that most TN terminations (49 (81.7%)) occurred above the MCA, which holds clinical significance in surgical approaches involving the tarsal tunnel and flexor retinaculum. All specimens (60/60, 100%) were preserved without any damage, ensuring the quality and reliability of the samples. To facilitate better visualization, 12 specimens were selectively colored with yellow paint. Furthermore, photographic documentation was consistently maintained for all specimens, ensuring thorough and accurate visual records for the study.

The most common pattern observed was type 1, the classical bifurcation into the MPN and LPN, seen in 54 (90%) out of 60 specimens. This result highlights a high degree of anatomical consistency in most of the population's branching pattern of the TN. In five (8.3%) specimens, a trifurcation pattern or an accessory calcaneal branch was noted. These variations are of clinical significance, particularly in foot surgeries (e.g., calcaneal procedures), tarsal tunnel syndrome, where accessory branches may be involved or compressed, and peripheral nerve blocks, where variant anatomy can affect the efficacy and safety of anesthetic delivery. In one (1.7%) specimen, no clear bifurcation was observed within the dissected field due to a high division occurring proximally, possibly above the dissection limit, or due to obscured visualization secondary to anatomical variation or cadaveric preservation issues. Type B variations emphasize the need for preoperative imaging or intraoperative nerve monitoring in select cases. Awareness of variant anatomy is essential for surgeons, anesthetists, and radiologists to avoid diagnostic errors and iatrogenic nerve injuries (Table 2).

**Table 2:** Morphological pattern of TN bifurcation Data are presented as numbers (N) and percentages (%). TN, tibial nerve

Pattern type	Description	Number of specimens (N=60)
Type A	Classical bifurcation (two terminal branches)	54 (90%)
Type B	Trifurcation or with accessory calcaneal branch	5 (8.3%)
Type C	No visible bifurcation within dissected area	1 (1.7%)

Most bifurcations occurred above the MCA, with a mean distance of 15.2±3.4 mm, confirming that in most specimens, the TN bifurcated within the tarsal tunnel, deep to the flexor retinaculum. These results have clinical significance in surgical decompression of the tarsal tunnel, where precise knowledge of this standard bifurcation level is critical to avoid nerve injury. Specimens terminating exactly at the MCA had a distance of 0 mm, corresponding to the inferior border of the flexor retinaculum. The rare cases where bifurcation occurred distal to the MCA showed a mean distance of 6.3±1.9 mm. These represent unusual branching patterns in which the division of the TN occurred beyond the tarsal tunnel (Table 3).

**Table 3:** Mean distance of MCA Data are presented as mean and standard deviation (SD). MCA, malleolar-calcaneal axis

Level of termination	Distance from MCA (mean±SD)
Above MCA	15.2±3.4 mm
At MCA	0 mm
Below MCA	6.3±1.9 mm

The TN bifurcation distribution pattern was comparable between the right and left lower limbs, indicating no significant lateral asymmetry in the termination level. This symmetry can be reassuring during comparative studies, bilateral clinical assessments, or surgical planning (Table 4).

**Table 4:** Comparison of bifurcation level between right and left limbs Data are presented as numbers (N) and percentages (%). MCA, malleolar-calcaneal axis

Level of termination	Right limb (n=30)	Left limb (n=30)
Above MCA	25 (83.3%)	24 (80%)
At MCA	4 (13.3%)	5 (16.6%)
Below MCA	1 (3.33%)	1 (3.33%

A single terminal bifurcation occurring near the MCA was observed in 55 (91.7%) out of 60 specimens with classical and most consistent anatomical patterns, where the TN divides into the MPN and LPN within or near the tarsal tunnel. The proximity to the MCA makes this a reliable anatomical landmark during surgical procedures and nerve blocks in the ankle region. In five (8.3%) specimens, a high division of the TN occurred proximal to the tarsal tunnel, possibly even in the posterior compartment of the leg. This variant can lead to the early branching of the MPN and LPN, which then pass individually through the tarsal tunnel. Such high divisions may be missed during nerve blocks if the anesthetic is injected only at the classical division site near the MCA (Table 5).

**Table 5:** Mode of termination of TN Data are presented as numbers (N) and percentages (%). MCA, malleolar-calcaneal axis; TN, tibial nerve

Mode	Number of specimens, N=60
Single terminal bifurcation (near MCA)	55 (91.7%)
High division (proximal to tarsal tunnel)	5 (8.3%)

## Discussion

This study aimed to determine the bifurcation level of the TN about the MCA and evaluate its clinical relevance. Dissection of 60 formalin-fixed cadaveric lower limbs revealed consistent anatomical patterns with noteworthy variations that have significant surgical and anesthetic implications.

In our study, the TN bifurcated above the MCA in 81.7% of specimens, aligning with the findings of Dellon and Mackinnon [9], who reported bifurcation within the tarsal tunnel in 95% of cases, and with Havel et al. [8], who observed similar results in 93% of specimens. These findings support using MCA as a reliable landmark in surgical interventions and regional anesthesia of the ankle and foot. Our results slightly exceed the 73% in-tunnel bifurcation reported by Louisia and Masquelet [11], suggesting possible population-based variability or methodological differences in defining tunnel boundaries.

A notable 15% of bifurcations occurred precisely at the MCA, a region considered the inferior edge of the flexor retinaculum. This transition zone is clinically significant, particularly during surgical decompression or nerve blocks, as bifurcation at this level may place both the MPN and LPN at risk.

Although rare, 3.3% of bifurcations were found below the MCA, indicating a more distal division of the TN. This finding challenges previous literature, such as Banik's [12] report of 100% bifurcation within the tarsal tunnel. It underscores the need for surgical caution in deep plantar approaches, where nerve branches may appear distal to their expected locations.

In 8.3% of specimens, we observed a high division of the TN proximal to the tarsal tunnel, possibly occurring in the posterior compartment of the leg. These early divisions resulted in MPN and LPN traversing the tunnel independently. Similar high divisions have been reported by Davis and Schon [13], who noted discrepancies between clinical examination and electromyography in such anatomical variants. These cases hold clinical significance in procedures like TN blocks, where anesthetic delivery solely near the MCA may miss more proximal bifurcations, leading to inadequate analgesia. While Kurtoglu et al. associated high divisions with accessory flexor digitorum muscles [14], no such muscular anomalies were found in our study; a larger sample may be needed for further evaluation.

Regarding the branching patterns, the classical bifurcation (type A) into the MPN and LPN was seen in 90% of specimens, consistent with Havel et al., who reported this pattern in 100% of cases [8]. Type B trifurcation or accessory branching, found in 8.3%, included additional medial calcaneal branches. This variation has been linked to entrapment syndromes such as tarsal tunnel syndrome, as noted by Warchol et al., [15] emphasizing the importance of identifying these branches during decompression procedures. A single case (1.7%) with no bifurcation observed within the dissected field (type C) may represent a high proximal division beyond the dissection field or an anatomical variant altered during preservation.

Quantitatively, the mean bifurcation distances were 15.2 mm above the MCA, 0 mm at the MCA, and 6.3 mm below, respectively. These results align with Banik’s study [12], which reported an average bifurcation distance of 1.86 cm above the MCA, reinforcing the MCA as a clinically relevant landmark for TN localization. These measurements are crucial for precise TN blocks, as advocated by Burton et al. [16], who reported their effectiveness in hallux valgus surgery and postoperative analgesia.

Laterality analysis showed no significant difference in bifurcation patterns between the right and left limbs, affirming the MCA's bilateral applicability as a surgical and anesthetic landmark.

The findings of this study affirm and expand upon previously published literature. While most bifurcations occurred proximal to or at the level of the MCA, the presence of high or low divisions and accessory branches highlights the importance of thorough anatomical understanding to minimize the risk of iatrogenic injury during surgical procedures and improve outcomes in regional anesthesia.

Limitations

Despite providing valuable anatomical insights, this study has several limitations. First, formalin-fixed cadaveric specimens may not fully replicate in vivo anatomical conditions due to potential tissue shrinkage or distortion, particularly of soft tissues like nerves and fascia. Second, the sample size was relatively limited (n=60 limbs) and derived from a single population, which may restrict the generalizability of the findings to broader or ethnically diverse populations. Third, laterality and sex-based differences were not analyzed in depth, which could have revealed subtle anatomical variations relevant to clinical practice. Additionally, no correlation with radiological imaging or clinical assessments was performed, limiting the translational applicability to surgical or anesthetic planning. Finally, the level of TN division was observed only up to the tarsal tunnel, so any bifurcation occurring more proximally in the leg may have been missed if it was beyond the dissection field.

Future studies using larger, more diverse samples, integration with imaging modalities, and in vivo validation may help overcome these limitations and refine the anatomical understanding of TN variations.

## Conclusions

The present study highlights the significance of the MCA as a reliable anatomical landmark for identifying the bifurcation of the TN. While the classical bifurcation proximal to the MCA is most commonly observed, recognition of higher divisions, trifurcations, and accessory branches is crucial for diagnostic accuracy and surgical intervention. These findings enhance the anatomical database specific to the Indian population and emphasize the importance of individualized surgical planning.

## References

[1] Malar D (2016). A study of tibial nerve bifurcation and branching pattern of calcaneal nerve in the tarsal tunnel. Int J Anat Res.

[2] Kim DI, Kim YS, Han SH (2015). Topography of human ankle joint: focused on posterior tibial artery and tibial nerve. Anat Cell Biol.

[3] Vasiliadis AV, Kazas C, Tsatlidou M, Vazakidis P, Metaxiotis D (2021). Plantar injuries in runners: is there an association with weekly running volume?. Cureus.

[4] Bilal F, Gamal G (2018). The tibal nerve (posterior tibal nerve): anatomical course and relation at the ankle. Open Acc Res Anatomy.

[5] Zhang Y, He X, Li J (2021). An MRI study of the tibial nerve in the ankle canal and its branches: a method of multiplanar reformation with 3D-FIESTA-C sequences. BMC Med Imaging.

[6] Torres AL, Ferreira MC (2012). Study of the anatomy of the tibial nerve and its branches in the distal medial leg. Acta Ortop Bras.

[7] Bage T, Power DM (2021). Iatrogenic peripheral nerve injury: a guide to management for the orthopaedic limb surgeon. EFORT Open Rev.

[8] Havel PE, Ebraheim NA, Clark SE, Jackson WT, DiDio L (1988). Tibial nerve branching in the tarsal tunnel. Foot Ankle.

[9] Dellon AL, Mackinnon SE (1984). Tibial nerve branching in the tarsal tunnel. Arch Neurol.

[10] Bilge O, Ozer MA, Govsa F (2003). Neurovascular branching in the tarsal tunnel. Neuroanatomy.

[11] Louisia S, Masquelet AC (1999). The medial and inferior calcaneal nerves: an anatomic study. Surg Radiol Anat.

[12] Banik S Sr, Guria LR (2021). Variable branching pattern of tibial nerve in the tarsal tunnel: a gross anatomical study with clinical implications. Cureus.

[13] Davis TJ, Schon LC (1995). Branches of the tibial nerve: anatomic variations. Foot Ankle Int.

[14] Kurtoglu Z, Uluutku MH, Can MA, Onderoglu S (2001). An accessory flexor digitorum longus muscle with high division of the tibial nerve. Surg Radiol Anat.

[15] Warchol Ł, Walocha JA, Mizia E, Bonczar M, Liszka H, Koziej M (2021). Ultrasound-guided topographic anatomy of the medial calcaneal branches of the tibial nerve. Folia Morphol (Warsz).

[16] Burton C, Sajja A, Latthe PM (2012). Effectiveness of percutaneous posterior tibial nerve stimulation for overactive bladder: a systematic review and meta-analysis. Neurourol Urodyn.

